# Transnasal Endoscopic Approach via Inferolateral Periorbital Periosteal Line for the Posterior Orbital Region

**DOI:** 10.1155/crot/3164871

**Published:** 2025-12-22

**Authors:** Teru Ebihara, Kazuhiro Omura, Iori Sano, Nobuyoshi Otori

**Affiliations:** ^1^ Department of Otorhinolaryngology, The Jikei University School of Medicine, Tokyo, Japan, jikei.ac.jp

**Keywords:** endoscopy, posterior orbit, transnasal

## Abstract

We report a minimally invasive transnasal endoscopic approach via the inferolateral periorbital periosteal line, which allows for safer surgery in the inferolateral orbital region without external incisions. This method enables better visibility while preserving physiological function and reducing cosmetic risks by minimizing fat deviation and avoiding problems such as ocular compression and orbital bone resection commonly encountered in open and transorbital surgery. This technique may be an optimal alternative or complement to traditional open surgery.

## 1. Introduction

The bony orbit is pyramidal in shape, with a rectangular base formed by the orbital rim and a narrow triangular apex at the optic canal and superior orbital fissure. Surgical intervention in the retroorbital region is particularly challenging because of its deep anatomical position and the complex network of adjacent blood vessels and nerves. Traditional surgical approaches to this area typically require an external incision; however, these methods frequently fail to provide adequate visualization and usually require lateral orbital bone resection. This increases the risk of postoperative complications, including injury to the extraocular muscles, ocular rupture, corneal anesthesia, intraorbital hematoma, and vision loss [1] by up to 35%.

With advancements in endoscopic techniques, transnasal endoscopic approaches for medial orbital wall and orbital floor fractures have gained widespread popularity in recent years. Furthermore, recent case series have demonstrated that endoscopic approaches can safely treat a wide spectrum of orbital pathologies with low complication rates and favorable cosmetic outcomes [2]. These techniques have been reported to be safe and effective, achieving high rates of improvement in conditions such as diplopia and enophthalmos, while maintaining a low incidence of complications [3].

Nevertheless, when dealing with the retroorbital region, these endoscopic approaches frequently encounter challenges, such as an insufficient field of view, the need for orbital traction, and limitations in the angle and maneuverability of the endoscope.

In 2024, Omura et al. identified a new anatomical landmark between the periorbital and periosteum of the posterior palatine nerve, located at the inferolateral periorbital periosteal line (ILPPL) [4]. Their cadaver study demonstrated that, by incising this junction, it was possible to reach several critical areas, including the posterior inferolateral orbital region, anterior medial temporal region, and lateral walls of the sphenoid and cavernous sinuses. This case report details the application of this novel ILPPL incision technique, combined with a prelacrimal approach [5] and a direct approach to the anterior and lateral part of the maxillary sinus with an endoscope (DALMA) [6] to treat a posterior lateral orbital fracture via a transnasal endoscope, providing a minimally invasive alternative to traditional external incisions.

## 2. Case Presentation

A 53‐year‐old male patient with no significant medical history presented to the hospital after sustaining head trauma due to a fall while intoxicated. His chief complaints were decreased vision in the left eye, pain during eye movement, and nausea. He was diagnosed with traumatic optic neuropathy, a left optic canal fracture, and a left zygomatic fracture. The patient was subsequently referred to our hospital for further treatment 24 h after the injury.

**Figure 1:**
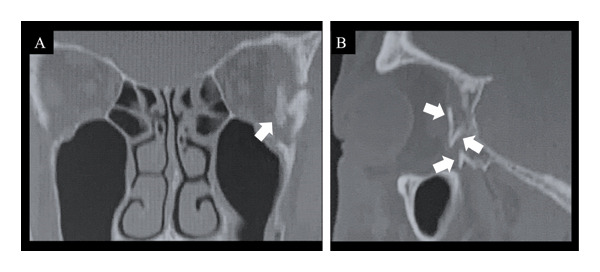
Preoperative CT image. (A) Coronal CT image. A posterior lateral orbital fracture is observed. (B) Sagittal CT image. Three bone fragments are observed posterior to the orbit. CT: computed tomography.

Physical examination revealed a contusion on the lateral side of the left eyebrow, accompanied by signs of internal bleeding around the left orbit. Computed tomography (CT) revealed fractures of the lateral wall of the left optic canal, the greater wing of the sphenoid bone extending into the orbit (Figure 1), and a left zygomatic fracture. The patient had no light perception in the left eye, and his eye movements were restricted in all directions. Given the severity of his symptoms, including pain and nausea, pulse steroid therapy was initiated. Surgical intervention was planned to decompress the orbit and remove bone fragments.

The surgery was performed under general anesthesia using a transnasal endoscope (Video S1) 26 h after the injury. The procedure included a prelacrimal approach and DALMA, which revealed fractures in the inferior orbital wall and the posterior wall of the maxillary sinus. The medial orbital wall and the fragmented orbital floor bone were carefully removed while preserving the orbital periosteum, thereby decompressing the orbit. In addition, the optic canal was decompressed, and the optic nerve was widely exposed and released.

**Figure 3:**
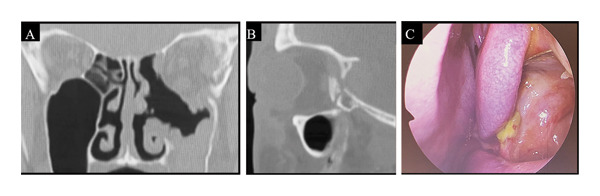
Postoperative CT and endoscopy image. (A, B) CT images taken 1 week postoperatively. All bone fragments had been removed, and decompression of the orbit had been achieved. (C) Left nasal 0‐degree endoscopy image taken 1 month after surgery. Epithelialization of the maxillary sinus was good. CT: computed tomography, ILPPL: inferolateral periorbital periosteal line.

The ILPPL, marking the boundary between the periosteum of the inferior orbital wall and the pterygopalatine fossa, was identified (Figures 2(a) and 2(b), white dotted line) and incised to allow access to the retroorbital space. The greater wing of the sphenoid bone (Figure 2(c), white arrow) was identified posteriorly. A hematoma and bone fragments were found in the lateral posterior–superior part of the orbit and were removed, with careful preservation of the surrounding tissues. Subsequently, the wound was covered with mucosa from the maxillary and sphenoid sinuses, and the incision was sutured to ensure the preservation of all turbinates and the edge of the piriform foramen. Postoperative CT confirmed the complete removal of the bone fragments and a successful orbital decompression without complications (Figures 3(A), 3(B)). Subsequent plastic surgery was performed to address the zygomatic and orbital floor fractures. One month postoperatively, visual function improved from no light perception to light perception at 1 m. Ocular motility also improved, with only a mild limitation in the leftward gaze remaining. Good epithelialization of the maxillary sinus was observed (Figure 3(C)). At 6 months postoperatively, light perception remained unchanged; however, the limitation in the leftward gaze had completely resolved.

Figure 2Incision approach to the inferolateral periorbital periosteal line. (a) Left 0‐degree endonasal endoscopy image. The posterior wall mucosa of the maxillary sinus is elevated to expose the inferior orbital periosteum and pterygopalatine fossa, and the inferolateral periorbital periosteal line (ILPPL, white dotted line) is observed between them. (b) Left 0‐degree endonasal endoscopy image. The ILPPL (white dotted line) is incised using a scalpel. (c) Left 0‐degree endonasal endoscopy image after incising the ILPPL. The greater wing of the sphenoid bone (white arrow) is observed immediately posteriorly. MT: middle turbinate, MS: maxillary sinus, PF: pterygopalatine fossa, SS: sphenoid sinus, ILPPL: inferolateral periorbital periosteal line.

**Figure:**
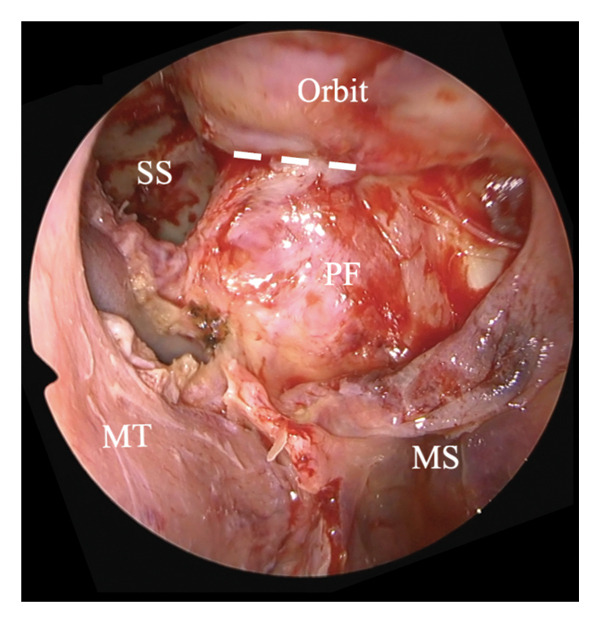
(a)

**Figure:**
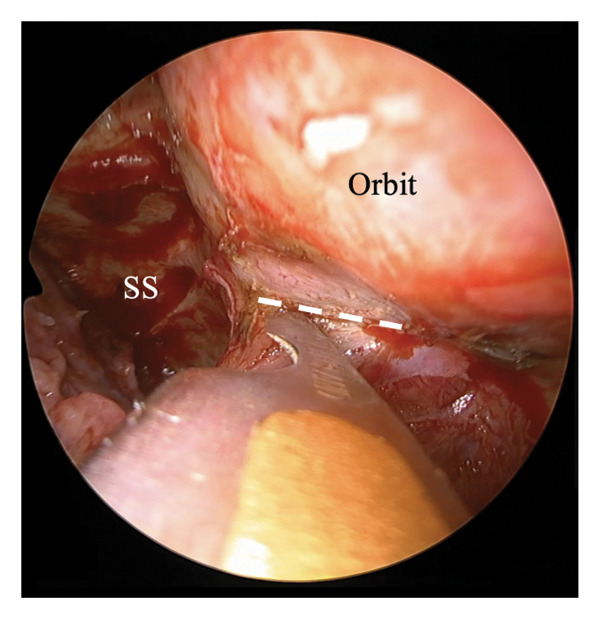
(b)

**Figure:**
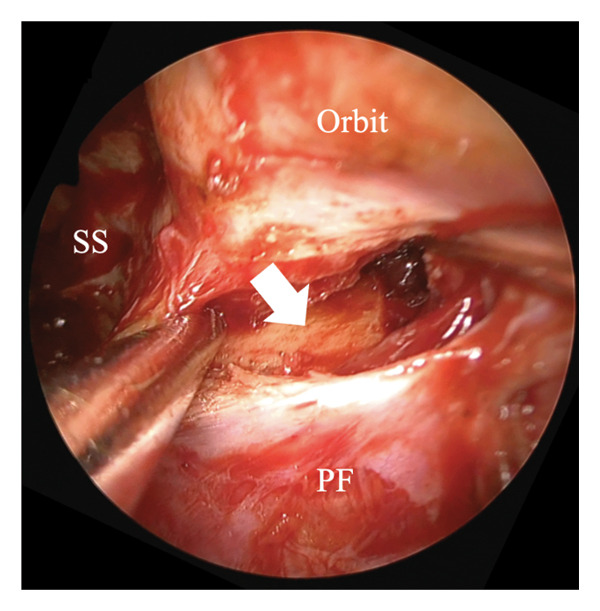
(c)

## 3. Discussion

The technical requirement for the transnasal endoscopic approach using the ILPPL incision is that the surgeon must be capable of performing the pterygoid approach. For surgeons familiar with the transpterygoid approach, this approach is a relatively simple procedure. Regarding the potential risks of this procedure, it does not require manipulation within the pterygopalatine fossa, including the maxillary artery; therefore, the overall risk is relatively low. However, since the maxillary strut is located between the infraorbital nerve and the superior orbital fissure, the size of the maxillary strut has individual variations. In case the maxillary strut is small, there are potential risks of injuries to this nerve and the contents of the superior orbital fissure. By combining the prelacrimal approach [5] with DALMA [6], it is possible to simultaneously insert multiple instruments in addition to an endoscope through the nose, which has a narrow and limited anatomical working space. Recent reviews have highlighted that techniques based on the prelacrimal approach [5] consistently achieve good disease control with preservation of the nasolacrimal duct and inferior turbinate, supporting our strategy of combining ILPPL incision with function‐preserving maxillary access [7]. This method maintains normal anatomical structure and physiological function while ensuring high operability. This advantage enables us to treat the retroorbital region, which is anatomically deep and has many surgical restrictions. Moreover, when approaching the pterygopalatine, multiple blood vessels are frequently sacrificed, and the middle and superior turbinates are often resected; however, our method does not require sacrifice, thereby preserving physiological functions.

The ILPPL incision approach has the advantage of minimizing the exposure of orbital fat and does not interfere with the surgical field. Eyeball compression by muscle retractors and orbital bone resection to secure the surgical field, which are problematic in open or transorbital approaches, are avoided, thereby allowing direct access to the posterior orbit. This enables surgery with a better visual field while minimizing the risk of cosmetic sequelae, such as extraocular muscle and nerve damage and enophthalmos. Furthermore, when the ILPPL is incised, the bone surface of the greater wing of the sphenoid bone is immediately reached, making the layers easy to understand and the surrounding landmarks clear. In the case of external incisions and transorbital approaches, the visual field angle and operating angle of the instruments are similar for the posterior orbit. Therefore, there are many limitations to the operation, and traction and compression of the eyeball are required. In our transnasal approach, the endoscope and instruments can be inserted at different angles, requiring minimal eyeball traction and compression. This technique has the advantage of minimizing eyeball damage. Regarding postoperative recovery, the ILPPL incision is approximately 2 cm, and the wound can be covered with the patient’s own mucosa, which is thought to result in good wound epithelialization and quick recovery while maintaining cosmetic and functional appearance.

Although the indications for this approach are limited to the posterior–inferior–lateral orbital region, it may represent an optimal approach that facilitates safe surgery with a better field of view by replacing the conventional external incision or transorbital approach or using it in combination with them.

## 4. Conclusion

Here, we report a novel, minimally invasive approach that uses a transnasal endoscope to incise the ILPPL and reach the posterior–inferior–lateral orbital region without the need for an external incision.

## Consent

Patient consent was obtained.

## Conflicts of Interest

The authors declare no conflicts of interest.

## Funding

No specific funding was obtained for this study.

## Supporting Information

Video S1: Transnasal endoscopic approach via inferolateral periorbital periosteal line.

The surgery was performed under general anesthesia using a transnasal endoscope (left nasal 0‐degree). The procedure included a prelacrimal approach and DALMA.

The ILPPL, marking the boundary between the periosteum of the inferior orbital wall and the pterygopalatine fossa, was identified (white dotted line) and incised to allow access to the retroorbital space. The greater wing of the sphenoid bone (white arrow) was identified posteriorly. A hematoma and bone fragments were found in the lateral posterior–superior part of the orbit and were removed, with careful preservation of the surrounding tissues. The wound was subsequently covered with mucosa from the maxillary and sphenoid sinuses, and the incision was sutured to ensure the preservation of all turbinates and the edge of the piriform foramen.

MT: middle turbinate, MS: maxillary sinus, PF: pterygopalatine fossa, SS: sphenoid sinus.

## Supporting information


**Supporting Information** Additional supporting information can be found online in the Supporting Information section.

## Data Availability

The data that support the findings of this study are available from the corresponding author upon reasonable request.
